# Inhibition of PIM1 attenuates the stem cell–like traits of breast cancer cells by promoting RUNX3 nuclear retention

**DOI:** 10.1111/jcmm.15272

**Published:** 2020-04-19

**Authors:** Hui Liu, Cheng Chen, Dongshen Ma, Yubing Li, Qianqian Yin, Qing Li, Chenxi Xiang

**Affiliations:** ^1^ Department of Pathology Xuzhou Medical University Xuzhou China; ^2^ Department of Pathology The Affiliated Hospital of Xuzhou Medical University Xuzhou China; ^3^ Laboratory of Clinical and Experimental Pathology Xuzhou Medical University Xuzhou China

**Keywords:** breast cancer, cancer stem cells, PIM1, RUNX3

## Abstract

Finding out the driver gene critical for the maintenance of breast cancer stem cells (BrCSCs) is important for designing a new strategy to eradicate these cells to improve patient's prognosis. Here, in our study, we revealed that PIM1, an oncogenic serine‐threonine kinase and a well‐proven contributor to the tumorigenesis of breast cancer, was involved in BrCSCs regulation and promised to be a new target for eradicating BrCSCs. In brief, PIM1 could enhance the stem cell–like traits of breast cancer cells by promoting the phosphorylation and cytoplasmic localization of RUNX3. The nuclear dislocation of RUNX3 disabled this tumour suppressor and led to breast cancer cells gaining stem cell–like traits. Inhibition of PIM1 significantly induced the nuclear retention of RUNX3, recovered its transcriptional function and attenuated the stem cell–like properties of breast cancer cells. Those findings deepened our understanding of PIM1's oncogenic effect, underlining the significance of PIM1 in designing a new strategy aimed at BrCSCs.

## 1 INTRODUCTION

Breast cancer is the most common cause of death in women worldwide. It was estimated that breast cancer will be diagnosed in 12% of women in the United States over the course of their lifetimes, and more than 250 000 new cases of breast cancer were diagnosed in the United States in 2017.^1^ Even now the mortality rate for breast cancer patients overall slowly declines with the improvement of early tumour detection and introduction of endocrine therapy in hormone receptor‐positive breast cancer, and triple‐negative breast cancer (TNBC) patients still hold the most dismal prognosis and more likely succumb to tumour relapse or metastasis, which usually ultimately lead to death. Chemical therapy is the only choice for patients suffering from TNBC or tumour metastasis.

Cancer stem cells (CSCs) are a group of cells resident within tumour bulk, however behaving differently from the rest of tumour cells.^2^ CSC population remain dormant when anticancer chemical agents attack the whole tumour bulk and after therapy withdrawn they initiate tumours by self‐renewal and differentiation, and spawn progenitors at a distant niche, leading to drug resistance, tumour relapse and metastasis.^3^ However, owing to the difficulty of isolating CSC population, few drugs are designed to specifically target at them. Hence, to figure out the underpinning biology of CSCs is important to develop new strategies to efficiently eradicate these cells, thus improving patient's prognosis.

PIM1 belongs to the PIM family of serine‐threonine kinases, which consists of three proto‐oncoproteins, PIM1, PIM2 and PIM3.^4^ The *pim*‐1 proto‐oncogene was first identified as a common proviral insertion site associated with murine leukaemia virus–induced lymphomagenesis, and its oncogenic activity was verified with transgenic mice overexpressing *Pim1* in the lymphoid compartment.^5^ The oncogenic roles of PIM1 were verified in solid tumours as colorectal cancer,^6^ hepatoma^7^ and gastric cancer.^8^ Knocking out all three PIM isoforms had limited side effects on mice,^9^ which suggested targeting at PIM kinases could be a new safe anti‐tumour strategy. PIM1 was reported to phosphorylate a variety of cell cycle‐controlling proteins thus enhancing cancer cell proliferation.^10^ In TNBC, PIM1 was shown to counteract the increased sensitivity to apoptosis induced by MYC activation.^7^, ^11^ However, the in‐depth oncogenic mechanism of PIM1 is not well‐elucidated, especially concerning its effect on breast cancer stem cells (BrCSCs).

RUNX3 belongs to the family of Runt‐related transcription factors (RUNX), and the RUNX family was identified to play a pivotal role in both normal development and neoplasia.^12^ RUNX3 was well identified to function as a tumour suppressor, and its inactivation was associated with tumorigenesis in lung adenocarcinoma, intestinal adenocarcinoma, colorectal cancer and gastric cancer.^12^, ^13^, ^14^, ^15^ In breast cancer, RUNX3 inactivation was reported to be related to tumorigenesis^16^ and YAP‐mediated stem cell–like traits.^17^ Cytoplasmic mislocation is an important mechanism by which RUNX3 loses its antitumour activity. RUNX3 can be phosphorylated by a spectrum of oncogenic kinases, like Pin1, Src, Pak1, to translocate from nucleus to cytoplasm, thus leading to its subcellular mislocation in human breast, pancreatic and gastric cancer.^18^, ^19^, ^20^ However in breast cancer, whether PIM1 acts as an upstream regulator of RUNX3 to phosphorylate it and promote its subcellular dislocation remains unclear and whether this mechanism plays a part in BrCSC‐regulating effect of RUNX3 is hardly referred before.

In this study, we revealed that inhibition of PIM1 kinase could attenuate the stem cell–like traits in breast cancer by rescuing the nuclear expression of RUNX3. We demonstrated that PIM1 could phosphorylate RUNX3 to facilitate its cytoplasmic retention, thus suppressing the transcriptional activity of RUNX3 and promoting breast cancer to gain BrCSC‐like traits. After PIM1 inhibition, RUNX3 could re‐localize to the nucleus and regain its anti‐BrCSC function. Moreover, RUNX3 was indispensable for the anti‐BrCSC effects of PIM1 inhibition. This finding suggested the important role of PIM1/RUNX3 axis in the regulation of BrCSC biology and offered new targets for eradicating BrCSC population.

## 2 MATERIALS AND METHODS

### 2.1 Tissue microarrays

Tissue microarray (TMA) blocks consisting of 213 breast cancer cases were obtained from Department of Pathology, The Affiliated Hospital of Xuzhou Medical University. TMA blocks were constructed following the clinical ethic guidelines. Ethics approval to perform this study was obtained from the Human Research Ethics Committee of the Xuzhou Medical Affiliated Hospital.

### 2.2 Immunohistochemistry (IHC) assay

Rehydrated slides taped from TMA block were boiled in antigen retrieval solution at 96°C for 40 minutes, then treated with serum‐free blocking solution (Beyotime) and incubated overnight at 4°C in a diluent solution (Beyotime) supplemented with monoclonal antibody targeting at RUNX3 (D236‐3, MBL, Japan) or PIM1 (sc‐374116, Santa Cruz, USA). A peroxidase‐3, 3′‐diaminobenzidine‐based detection system (Zsbio) was used to detect the immunoreactivity. H‐score was calculated by multiplying the staining intensity (ranged from 0 to 3) with 100× percentage of positively stained area to obtain a number scaled 0‐300. The scoring was performed by a single pathologist (NS) following consultation with another pathologist (MST) and in the absence of any clinical information informed.

The detection of CD44 and CD24 on a same slide was performed according to the instructions of Polymer Doublestain Kit (ZSGB‐BIO). CD44 (Clone 156‐3C11, 1:200) (Invitrogen) was detected with diaminobenzidine (DAB) and CD24 (Clone SN3b, 1:100) (Invitrogen) with Permanent Red. The proportion of CD44+/CD24− BrCSCs^21^ was determined as the percentage of cells positive for DAB staining but negative for Permanent Red staining.

### 2.3 Immunofluorescence (IF) assay

The immunofluorescence assay was conducted as described.^22^ In brief, slides were fixed in 4% paraformaldehyde and blocked with 5% BSA, followed by incubation with anti‐PIM1 or anti‐RUNX3 antibody in blocking solution at 4°C overnight. Wash the slides using 1 × PBS (0.1% Tween‐20) for 3 times and incubate them in blocking solution with goat anti‐rabbit IgG 488 or goat antimouse IgG 549 for 30 minutes, followed by DAPI for another 5 minutes. Slides were mounted and imaged with a fluorescence microscope (ZEISS AXIO, Germany). The quantification of immunofluorescence intensity was conducted in Image J_v1.8.0 by measuring the staining intensity of cytoplasm or nucleus for every single cell and calculating the ratio cytoplasm/(cytoplasm + nucleus) or nucleus/(cytoplasm + nucleus). Data were presented in triplicate by measuring three images randomly captured from different fields.

### 2.4 Cell culture

The breast cancer cell lines T47D, MCF‐7, SK‐BR‐3, BT‐549 and MDA‐MB‐231, as well as non‐malignant MCF‐10A cells, were stored in our laboratory. All cell lines were maintained as recommended by the suppliers. The cell lines were authenticated by short‐tandem‐repeat (STR) analysis every year. All cell lines were cultured following standard procedure.

### 2.5 Cell transfection

Transfection of plasmids or siRNA was performed using Lipofectamine 2000 (Invitrogen) according to the product guidance. A final concentration of siRNA (GenePharma) was 50 nmol/L, and concentration of DNA was 4 μg/mL. Sequences of siRNA against specific target were listed below:

RUNX3 siRNA sequence:

Sense, 5′‐UGACGAGAACUACUCCGCUTT‐3′,
Antisense, 5′‐AGCGGAGUAGUUCUCGUCATT‐3′;
PIM1 siRNA sequence:
Sense, 5′‐CCAUCAAACACGCACGUGGAGAATT‐3′,
Antisense, 5′‐UUCUCCACGUGUUUGAUGGTT‐3′.

### 2.6 RNA isolation and quantitative real‐time PCR (qRT‐PCR) analysis

Total RNA was extracted by RNA isolator reagent (Vazyme) according to the manufacturer's instructions. cDNA was generated using All‐In‐One RT MasterMix (ABM) according to the manufacturer's instructions. Real‐time PCR was carried out with ABI Step One Plus Real‐Time PCR system (ABI) using primers specific for the detection of Cdkn1a (p21), and the values were normalized relative to the expression of endogenous control gene GAPDH. Sequences of primers used for qRT‐PCR in this study are listed as below:

Cdkn1a (p21) (GenBank accession: NM_078467):
Forward primer, 5′‐TGTCCGTCAGAACCCATGC‐3′,
Reversed primer, 5′‐AAAGTCGAAGTTCCATCGCTC‐3′;
GAPDH (GenBank accession: NM_001256799):
Forward primer, 5′‐CTGGGCTACACTGAGCACC‐3′,
Reversed primer, 5′‐AAGTGGTCGTTGAGGGCAATG‐3′.

### 2.7 cDNA expression constructs

The complete PIM1 protein coding sequence (NCBI reference sequence NM_001243186.1) was subcloned into the pCMV‐N‐myc vector (Beyotime, China). The complete RUNX3 cDNA sequence (NCBI reference sequence NM_004350.2) was subcloned into the pCMV‐N‐FLAG vector (Beyotime, China). A mutant RUNX3(RUNX3(4A)) construct was generated by introducing site‐specific mutations into the complete *RUNX3* coding sequence (pCMV‐N‐FLAG vector), by substituting Ser149, Thr151, Thr153 and Thr155 into alanine, under the guidance of PCR‐based site‐directed mutagenesis kit (Vazyme, China).

Primers to generate coding sequence of PIM1:

Forward primer, 5′‐CCCAAGCTTATGCTCTTGTCCAAAATCAACTCGC‐3′,
Reversed primer, 5′‐CGGAATTCCTATTTGCTGGGCCCCGGC‐3′;
Primers to generate coding sequence of RUNX3:
Forward primer, 5′‐CCCAAGCTTATGCGTATTCCCGTAGACCCAAG‐3′,
Reversed primer, 5′‐CGGAATTCTCAGTAGGGCCGCCACACG‐3′;
Primers to generate RUNX3 (4A) were designed following the instruction:
Forward primer, 5′‐GGCTTTCGCCCTGGCCATCGCTGTGTTCACCAACCCCACCCAA‐3′, reversed primer, 5′‐GCGATGGCCAGGGCGAAAGCCTTCCCTCGCCCACTGCGG‐3′.

### 2.8 Western blot (WB) analysis and Immunoprecipitation (IP)

Cells were lysed by scraping them into RIPA lysis buffers supplemented with 1% PMSF. The cell lysates were maintained on ice with vortex every 10 minutes and then subjected to centrifuge at 4°C, 11 000 *g*. The supernatant was harvested for BCA protein assay (Beyotime, China). In some cases, nuclear and cytoplasmic proteins were separated using Nuclear and Cytoplasmic Protein Extraction Kit (Beyotime).

Primary antibodies used in this study were listed below: RUNX3 (ab40278, Abcam); PIM1(sc‐374116, Santa Cruz, USA); GAPDH (10494‐1‐AP, Proteintech); p21(10355‐1‐AP, Proteintech); NANOG (14295‐1‐AP, Proteintech); OCT‐3/4 (wl01728, Wanleibio); histone‐H3 (17168‐1‐AP, Proteintech); SOX2 (11064‐1‐AP, Proteintech).

Immunoprecipitation was performed following standard procedure. Specially, cytoplasmic protein was acquired using Nuclear and Cytoplasmic Protein Extraction Kit (Beyotime) and subjected to immuoprecipitation with Protein A/G PLUS‐Agarose (sc‐2003, Santa Cruz, USA) and anti‐PIM1 antibody (sc‐374116, Santa Cruz, USA) or mouse IgG (Beyotime) as negative control.

Dephosphorylation of PIM1 was performed according to the manufacturer's instructions. Incubate 200 μL cell lysates with 5 μL Lamda protein phosphatase (λ‐PPase) (NEB, USA) at 30°C for 30 minutes. The cell lysates were then subjected to immunoprecipitation by anti‐PIM1 antibody (Santa Cruz, USA), and the reactions were stopped by 2× Laemmli sample buffer (BD, USA) at 70°C for 10 minutes.

### 2.9 Flow cytometric assay

Flow cytometric assay was conducted following standard procedure. In brief, single‐cell suspension (10^6^ cells/mL) were acquired by Accutase (Invitrogen) digesting, resuspended (10^6^ cells/mL) in running buffer (1 × PBS, 0.5% BSA and 5 mmol/L EDTA) supplemented with FITC‐conjugated human CD44 antibody (Biolegend) and APC‐conjugated human CD24 antibody (Biolegend) and finally subjected to flow cytometer (BD Verse) for detecting CD44+/CD24− population. Data were analysed by FlowJo X.

### 2.10 ALDH1 activity assay

ALDH1 activity of breast cancer cells was assessed by ALDEFLUOR kit (STEMCELL) according to the instruction provided by the manufacturer. In brief, adjust the cell sample to a final concentration of 10^6^ cells/mL. 5 μL activated ALDEFLUOR™ Reagent was added to 1 mL cell suspension (test tube) and immediately transfer 0.5 mL to another tube containing 5 μL ALDEFLUOR™ DEAB Reagent (Control tube). Incubate “Test” and “Control” tubes for 30‐60 minutes at 37°C. Following incubation, centrifuge all tubes for 5 minutes at 250 *g* and remove the supernatant. Resuspend the cell pellet in 0.5 mL ALDEFLUOR™ Assay Buffer and subject the cell suspension to flow cytometric analysis.

### 2.11 Luciferase reporter assay

The promoter region of Cdkn1a (the sequence was from Genecopoeia) was cloned into pGL3‐basic vector. Nonsense sequence control siRNA (NC) or siPIM1 was co‐transfected with pGL3‐Cdkn1a and Renilla luciferase control plasmid by Lipofectamine 2000 (Thermo Fisher). The assay was conducted in triplicate following the instruction of Dual Luciferase Reporter Assay System (Promega) 48 hours after transfection, with Firefly luciferase activity being normalized to Renilla luciferase activity.

### 2.12 3D Mammosphere formation assay

Cells were detached from plates by Accutase (Invitrogen) and resuspended in MammoCult medium (Stem Cell Technologies). The single‐cell suspension prepared was mixed with methylcellulose and re‐plated in 24‐well ultra‐low attachment plate (Corning). The mammosphere colony formed after 2‐week culture in a CO_2_ incubator. The colony numbers were counted, and the diameters of the colony were measured under an inverted microscope (Olympus).

### 2.13 Clonogenic assay

Detach cells from the tissue culture plate using 0.25% Trypsin‐EDTA solution and resuspend cells in serum‐free cell culture media to a final concentration of 10^6^ cells/mL. An appropriate number of cells (3000‐5000) were seeded per chamber on a 24‐well plate. Incubate the plates in a CO_2_ incubator at 37°C for 1 week until cells in control group have formed colonies with substantially good size. Add 1 mL 4% paraformaldehyde fix solution per well and leave the plates at room temperature (RT) for 5 minutes. Remove fixation solution, and add 1 mL 0.5% crystal violet solution per well and incubate the plate at RT for 2 hours. Remove crystal violet carefully and immerse the plates in distilled water to rinse off extra crystal violet. Air‐dry the plates and photograph the colonies by a Zeiss microscope.

### 2.14 Proliferation assay

After transfection for 24 hours, cells were detached from plates by 0.25% Trypsin‐EDTA solution and resuspend cells in serum‐free cell culture media to 106 cells/mL. 3000‐5000 cells were seeded per chamber on a 96‐well plate. Incubate the plates in a CO_2_ incubator at 37°C for another 48 hours. 10 μL CCK8 was added to each cell and incubate the plate for 3 hours. Microplate reader (Thermofisher) detected the absorbance at 450 nm. After deducting the blank, the proliferation rate of cells was calculated relative to control group.

### 2.15 Cell invasion assay

Cells are detached from the tissue culture plate using a 0.25% Trypsin solution and resuspend cells in serum‐free cell culture media containing 0.1% BSA (bovine serum albumin). Re‐adjust the cell suspension to a concentration of 10^6^ cells/mL. Matrigel (BD, #356234) is thawed and liquefied on ice, and then, 30‐50 μL of Matrigel is added to a 24‐well Transwell insert (Corning) and solidified in a 37°C incubator for 30 minutes to form a thin gel layer. After that, 200 μL cell suspension is added to the upper well, and 500 μL cell culture media containing 10% FBS is added to the bottom well to stimulate the cells in the upper chamber to translocate through the extracellular matrix and the membrane. Incubate the plates in a CO_2_ incubator at 37°C for 24‐48 hours. Remove the Transwell insert from the plate and carefully erase the cells that have not migrated through the membrane. Place the Transwell insert into 4% paraformaldehyde fix solution for fixation for 10 minutes and then 0.2% crystal violet for staining for 5 minutes. Remove the excess crystal violet, wash the insert with PBS for 3 times, air‐dry the Transwell inserts and photograph the colonies on the Transwell membrane by a Zeiss microscope. The quantification of Transwell assay results is conducted in Image J_v1.8.0 by measuring the crystal violet‐stained area of each group.

### 2.16 Statistical analysis

All data were obtained from at least three independent experiments (n ≥ 3) and presented as the mean ± SD (standard deviation). Statistical analyses (two‐tailed *t* test, Pearson correlation, chi‐square test, survival analyses, log‐rank test and Tukey's multiple‐comparison test) were performed using Prism 6 (version 6.0f) from GraphPad Software, Inc Contingency table and progression‐free survival (PFS) were also created and analysed using Prism 6 (version 6.0f). *P* < .05 was considered to indicate statistical significance throughout this study.

## 3 RESULTS

### 3.1 PIM1 facilitated the stem cell–like traits in breast cancer cells

**FIGURE 1:**
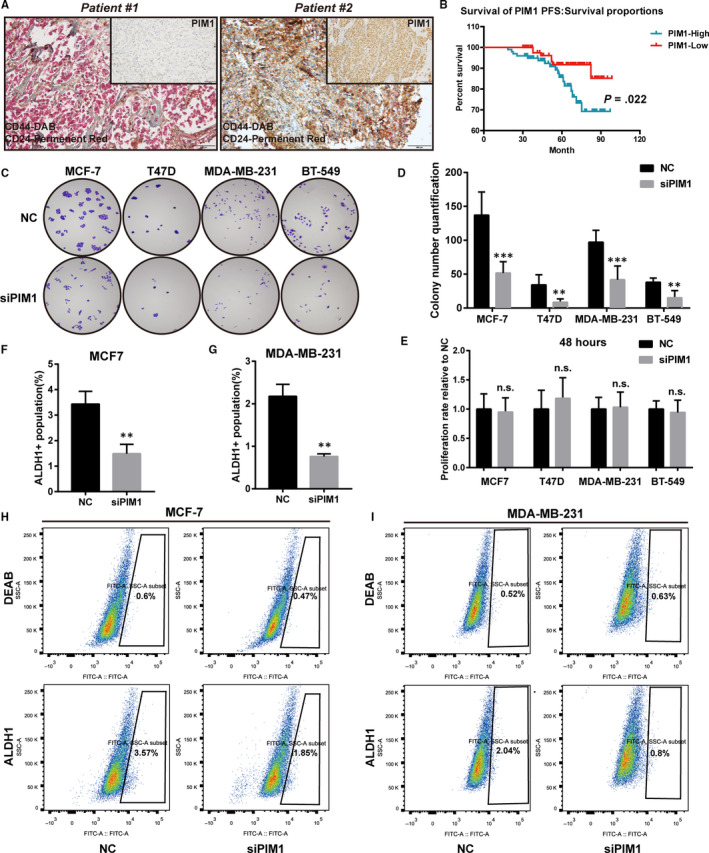
PIM1 facilitated the stem cell–like traits in breast cancer cells. A, Representative cases of IHC staining in the TMA with CD44 and CD24 double‐stained in a same slide. Note that in Patient 1 whose PIM1 was absent, most cancer cells were CD44− and CD24+, whereas in Patient 2 with pervasive expression of PIM1, most cancer cells were CD44+ and CD24−. B, PFS curve stratified by PIM1 expression. High PIM1 expression significantly indicated a worse progression‐free survival (*P* = .022). C, Representative images of clonogenic assay showing that PIM1 knockdown significantly reduced the clonogenic ability of breast cancer cells (n = 4). D, The quantification of colonies formed in (C). E, CCK8 assay showing that PIM1 knockdown did not significantly impair the proliferation of breast cancer cells within 48 h (n = 6). F and G, The statistical analysis of ALDH1‐positive population in H (F) and I (G). H and I, Representative images of flow cytometric assays showing that PIM1 knockdown reduced ALDH1+ population of MCF‐7 (H) and MDA‐MB‐231 (I) (n = 3). Statistical significance was assessed using unpaired Student's *t* test and one‐way ANOVA (***P < .001; **P < .01; and *P < .05; n.s., no significance.)

It was well mentioned that PIM1 played pivotal roles in the tumorigenesis of breast cancer. As expected, PIM1 was pervasively expressed across breast cancer cell lines irrespective of hormone receptor status with the exception of MCF‐10A, a normal human breast epithelial cell line, which barely expressed PIM1 (Figure S1A). We also performed IHC staining on a TMA consisting of 213 breast cancer cases. The expression of PIM1 was not correlated with conventional clinicopathological parameters such as age, tumour subtype, histological grade, clinical stage or Ki67 expression. However, high PIM1 expression was related to worse PFS and a high proportion of BrCSC population (CD44+/CD24−) (Table 1, Figure 1A,B).

**TABLE 1:** Contingency table of PIM1 expression, BrCSC proportion and other clinicopathological parameters

Variables	n	PIM1 Expression		*χ* ^2^	*P*
		PIM1‐Low	PIM1‐High		
Age (y)					
<48	89	46	43	1.377	.241
≥48	124	54	70		
Subtype					
Lumina A	43	25	18	5.120	.163
Lumina B	49	26	23		
Her2 positive	44	19	25		
TNBC	77	30	47		
Histological grade					
I	32	18	14	1.684	.641
II	140	65	75		
II	39	16	23		
IV	2	1	1		
Clinical stage					
I + II	178	86	92	0.812	.368
III + IV	35	14	21		
Ki67 expression					
<30%	53	33	20	2.168	.141
≥30%	160	81	79		
BrCSC (CD44+/CD24−)					
<10%	161	82	79	4.025	.045
≥10%	54	19	35		

To elucidate the contribution of PIM1 to BrCSCs, we used clonogenic assay to evaluate the impact of PIM1 inhibition on the ability of breast cancer cells to form colonies. As shown in Figure 1C,D, the clonogenic ability was reduced when knocking down PIM1 using siPIM1. However, knocking down PIM1 did not affect the proliferation rate, indicating the impaired colonies formation ability after PIM1 knockdown was not due to inhibited cells growth (Figure 1E). We then assessed the ALDH1 activity following PIM1 knockdown using flow cytometric assay. As expected, knockdown of PIM1 significantly down‐regulated ALDH1+ population in MCF‐7 (Figure 1F,H) and MDA‐MB‐231 (Figure 1G,I) cells.

### 3.2 RUNX3 mediated the anti‐BrCSC effects of PIM1 inhibition

PIM1 was revealed to be able to phosphorylate RUNX3 at Runt region of RUNX3, promoting RUNX3 to translocate from nucleus to cytoplasm,^23^, ^24^ and RUNX3 was recently reported as negative regulator of BrCSCs.^17^ We then hypothesized that RUNX3 mediated the anti‐BrCSC effect of PIM1 inhibition. RUNX3 was substantially expressed in the cytoplasm of breast cancer cell lines while in the nucleus of normal breast epithelial cell line MCF‐10A (Figure S1A‐C). Indeed, RUNX3 was shown to attenuate the stem cell–like traits of breast cancer cells: facilitating RUNX3 expression in both MCF‐7 and MDA‐MB‐231 cells led to reduced mammosphere formation capacity, indicated by reduced mammosphere forming frequencies and smaller sphere diameters; conversely, knock down of RUNX3 in MCF‐7 and MDA‐MB‐231 cells promoted the stem cell–like traits of breast cancer cells, indicated by more spheres formed and their larger size (Figure S2A‐C). Moreover, the expression of stemness‐related transcriptional factors (ie Nanog, Oct‐4 and Sox2) was confirmed to be attenuated by overexpressing RUNX3 and promoted by knocking down RUNX3 (Figure S2D,E).

**FIGURE 2:**
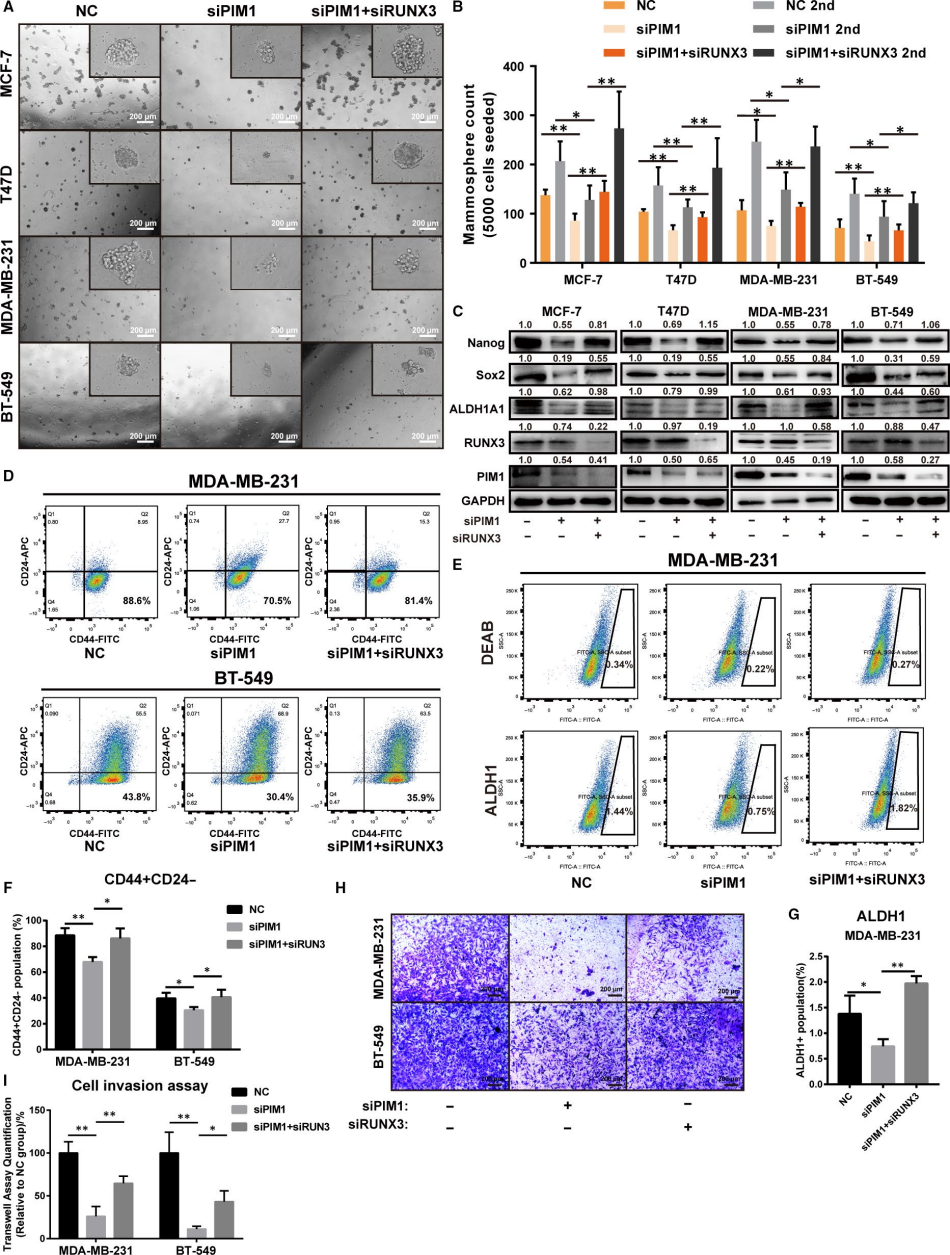
RUNX3 mediated the anti‐BrCSC effect of PIM1 inhibition. A, Representative images of mammosphere formation assay (the first generation) showing that PIM1 knockdown significantly reduced the mammosphere formation capacity while additionally RUNX3 knockdown reversed it. B, Quantification of mammospheres formed in (A). C, Western blot analysis showing that PIM1 knockdown reduced the expression of stem cell–related transcriptional factors and this was reversed by additionally RUNX3 knockdown in MCF‐7, T47D, BT‐549 and MDA‐MB‐231 cells. D, Representative images of flow cytometric analysis showing the CSC population gated by CD44+ and CD24−. E, Representative images of ALDH1 positive population gated out with DEAB treatment group as negative control. F, Quantification of CD44+ CD24− population in (D) (n = 3). G, Quantification of ALDH1 positive population in (E) (n = 3). H, Cell invasion assay showing that cell invasion was significantly reduced after PIM1 knockdown and was reversed when additionally knocking down RUNX3. I, Quantification of Transwell assay in (H) (n = 3). Data were expressed as means ± SE of three independent experiments. Statistical significance was assessed using unpaired Student's *t* test and one‐way ANOVA (***P < .001; **P < .01; and *P < .05)

We next sought to confirm whether RUNX3 could mediate the anti‐BrCSC effect of PIM1 knockdown. In line with the aforementioned results, after knocking down PIM1 in MCF‐7, T47D, MDA‐MB‐231 and BT‐549, their mammosphere formation capacity was significantly dampened and then rescued by knocking down RUNX3 additionally (Figure 2A,B). The expression of stemness‐related proteins Nanog, Sox2 and ALDH1A1 was significantly inhibited by knocking down PIM1 with siPIM1, and this effect exerted by siPIM1 was reversed by knocking down RUNX3 simultaneously (Figure 2C), suggesting that RUNX3 mediated the stemness‐attenuating effects of PIM1 inhibition. Moreover, down‐regulated CD44+/CD24− and ALDH1+ population by siPIM1 was reversed by knocking down RUNX3 simultaneously (Figure 2D‐G). Interestingly, the invasion capacity of breast cancer cells was also reduced by knocking down PIM1 and the anti‐invasion effect of siPIM1 could also be attenuated by additionally knocking down RUNX3 (Figure 2H,I). All these results suggested the anti‐BrCSC effects of PIM1 inhibition, and the indispensable role of RUNX3 in this process.

**FIGURE 3:**
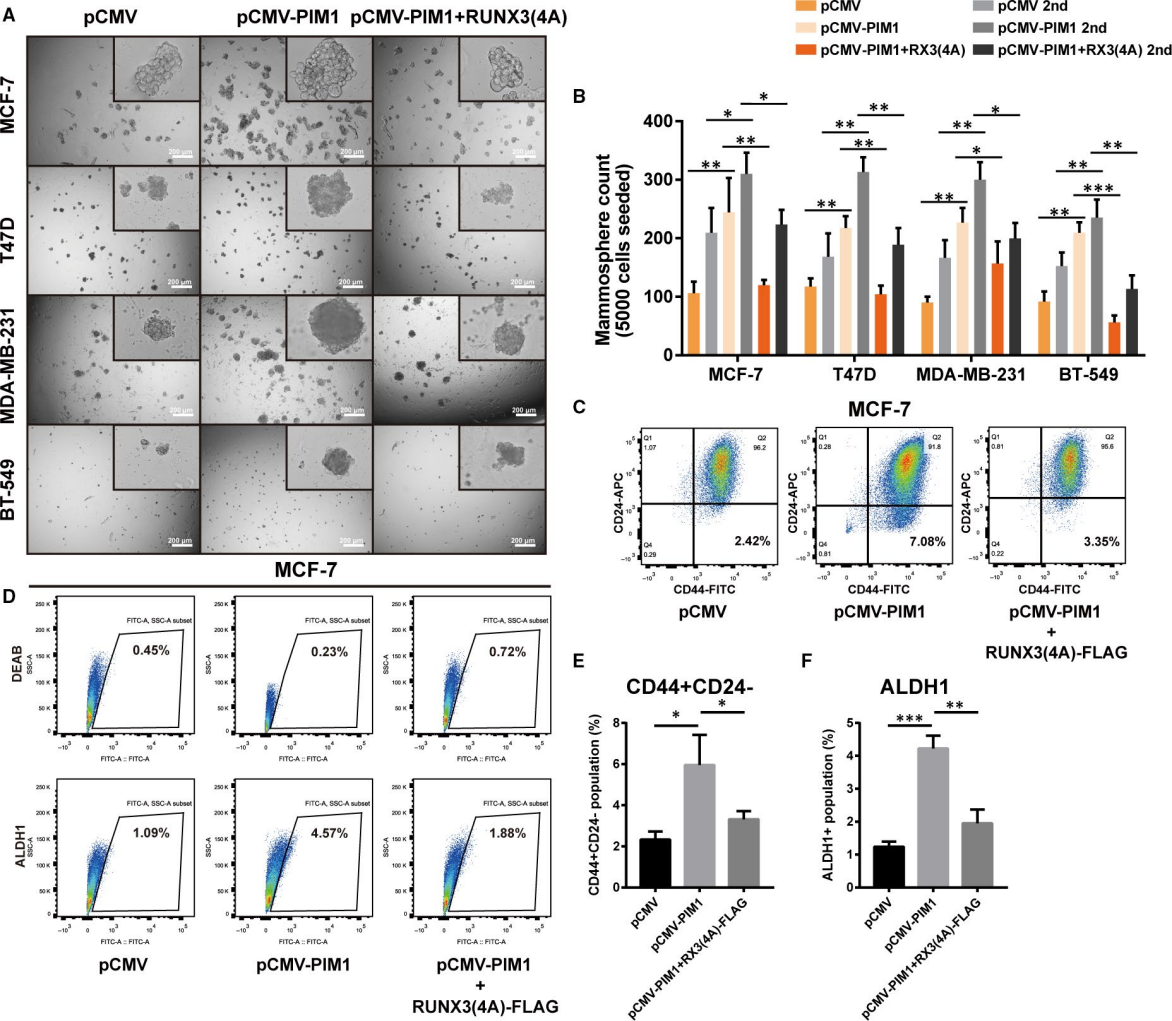
RUNX3(4A) reversed the up‐regulation of stem cell–like traits induced by PIM1. A, Representative images of mammosphere formation assay (the first generation) showing that ectopic PIM1 expression significantly promoted the mammosphere formation capacity while additionally RUNX3(4A) overexpression reversed it (n = 3). B, Quantification of mammospheres formed in (A). C and D, Representative images of flow cytometric assay detecting CSCs population (CD44+ CD24− in [C] and ALDH1+ in [D]) (n = 3). Noting that overexpression of PIM1 increased the CSCs population; however, simultaneous overexpression of RUNX3(4A)‐FLAG reversed it. E and F, Quantification of data in C (E) and D (F). Data were expressed as means ± SE of three independent experiments. Statistical significance was assessed using unpaired Student's *t* test and one‐way ANOVA (***P < .001; **P < .01; and *P < .05)

Moreover, we tried to further confirm the possible role of PIM1 in the gaining of stem cell–like traits for breast cancer cells. As expected, facilitating PIM1 expression in breast cancer cells (MCF‐7, T47D, MDA‐MB‐231 and BT‐549) led to elevated mammospheres formation capacity and that was reduced by additionally overexpression of RUNX(4A)‐FLAG, which is deficient of the phosphorylation sites targeted by PIM1 (Figure 3A,B). BrCSCs population (CD44+/CD24− and ALDH1+) was also expanded after PIM1 overexpression in MCF‐7 cells and this was reversed by additionally RUNX3(4A) overexpression (Figure 3C‐F). In conclusion, all these results suggested that PIM1 could promote breast cancer cells to gain stem cell–like traits and inhibiting PIM1 promised to exert anti‐BrCSC effects with RUNX3 being indispensable in this process.

### 3.3 RUNX3 attenuated the epithelial‐mesenchymal transition (EMT) mediated by PIM1 overexpression

**FIGURE 4:**
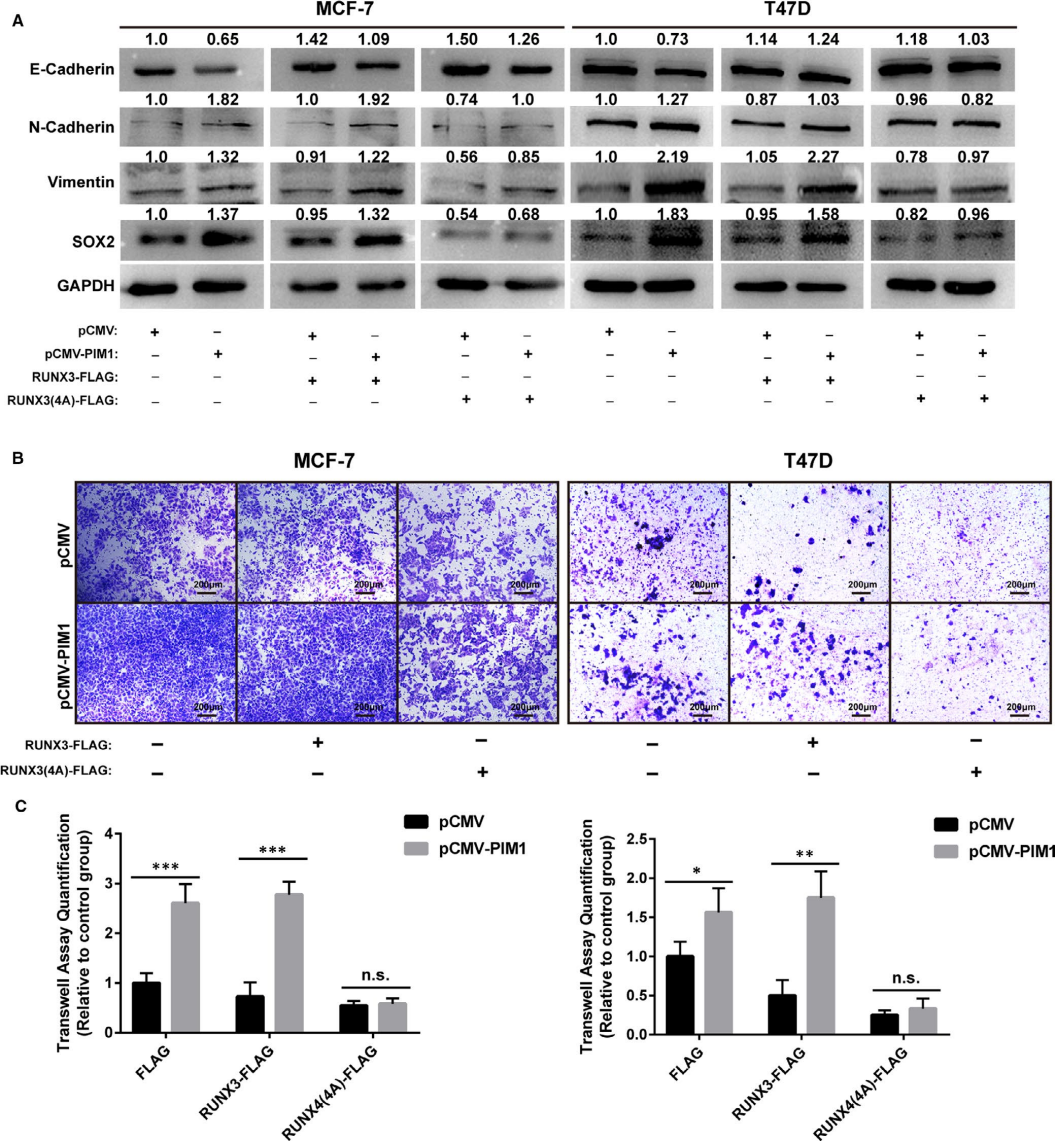
RUNX3(4A) attenuated the EMT induced by PIM1. A, Western blot showing that overexpression of PIM1 down‐regulated the expression of epithelial genes and up‐regulated that of mesenchymal genes. RUNX3(4A) but not RUNX3 could abolish the pro‐EMT effect of PIM1 overexpression. B, Transwell assay showing that overexpression of PIM1‐induced cell invasion (n = 3). RUNX3(4A) but not RUNX3 could abolish the pro‐invasion effect of PIM1 overexpression. C, The quantification of data in B. Data were expressed as means ± SE of three independent experiments. Statistical significance was assessed using unpaired Student's *t* test and one‐way ANOVA (***P < .001; **P < .01; and *P < .05)

Cell invasion and EMT were often intricately related to stem cell–like traits of cancer cells. We thus sought to investigate the role of PIM1 and RUNX3 in EMT process of breast cancer cells. Overexpression of PIM1 significantly down‐regulated epithelial marker E‐cadherin but up‐regulated mesenchymal marker N‐cadherin, vimentin and also the stem cell–related transcriptional factor SOX2 (Figure 4A). These changes were reversed by overexpression of RUNX3(4A)‐FLAG while only slightly attenuated by overexpression of RUNX3‐FLAG. In line with this, Transwell assay showed that overexpression of PIM1 could promote the cell invasion capacity whereas overexpression of RUN3‐FLAG(4A) but not RUNX3‐FLAG reversed it (Figure 4B,C). All these results suggested that PIM1 could promote the EMT process of breast cancer cells and this effect could be attenuated by RUNX3 and reversed by RUNX3(4A). And this was in accordance with the anti‐invasion effect of PIM1 knockdown and also the further pro‐invasion effect of RUNX3 knockdown (Figure 2F). We also noted that RUNX3(4A), which could not be phosphorylated by PIM1, more profoundly reversed the pro‐EMT effects of PIM1. And this implied us that PIM1 might exert both its pro‐BrCSC and pro‐EMT effects by phosphorylating RUNX3 due to the fact that breast cancer cells could gain stem cell–like traits by undergoing EMT process.^25^, ^26^, ^27^

### 3.4 PIM1 phosphorylated RUNX3 to promote its nuclear exportation

**FIGURE 5:**
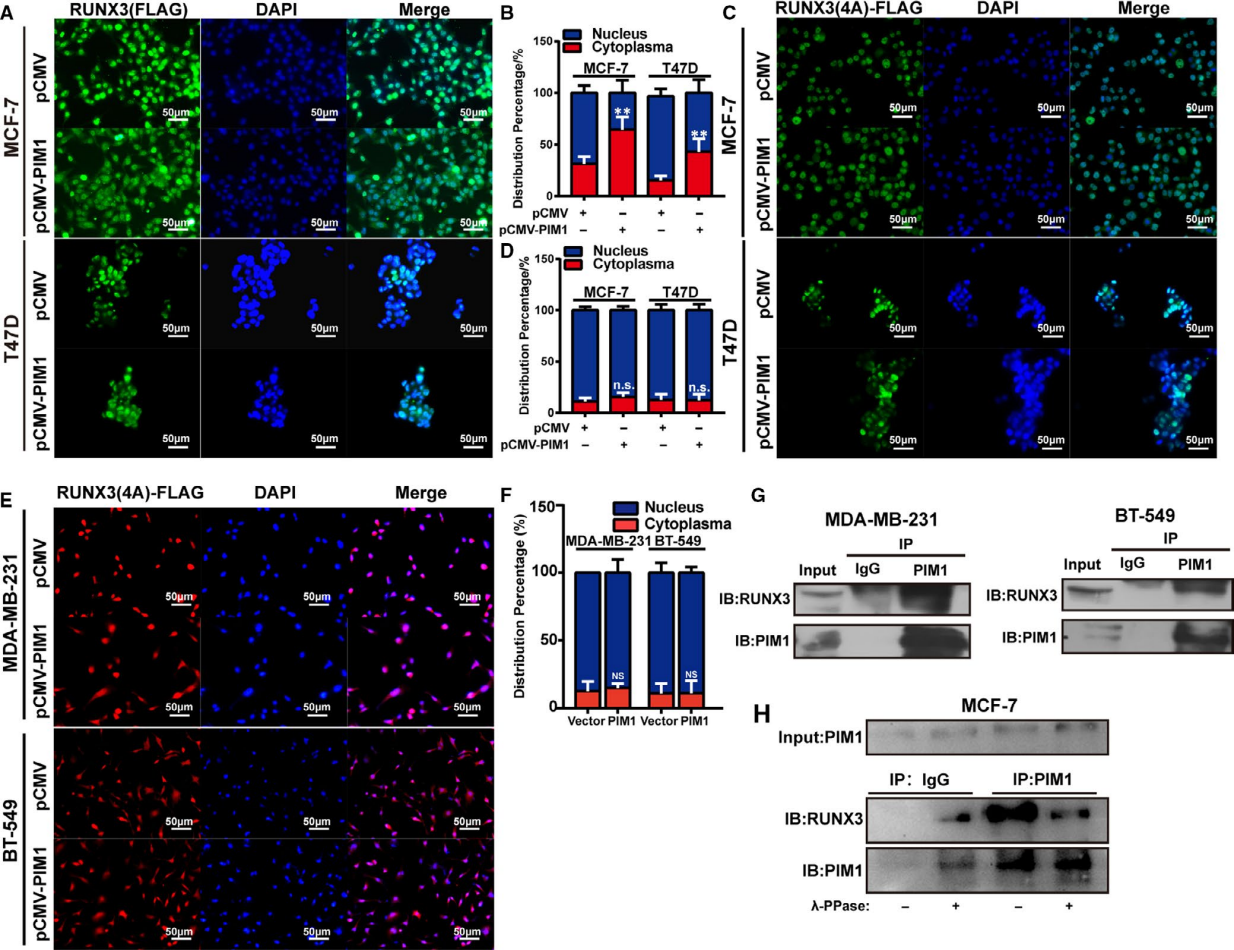
PIM1 facilitated RUNX3 cytoplasmic localization by directly interacting with and phosphorylating RUNX3. A, Immunofluorescence images showing the localization of ectopic wild‐type RUNX3 in MCF‐7 and T47D cells transfected with pCMV‐N‐myc vector plasmids or pCMV‐N‐myc‐PIM1. B, indicated the percentage of nuclear/cytoplasmic expression of RUNX3 in A. C, Immunofluorescence images showing the localization of RUNX(4A) in MCF‐7 and T47D cells transfected with pCMV‐N‐myc vector plasmids or pCMV‐N‐myc‐PIM1. D, indicated the percentage of nuclear/cytoplasmic expression of RUNX3 in C. E, Immunofluorescence images showing the localization of RUNX(4A) in BT‐549 and MDA‐MB‐231 cells transfected with pCMV‐N‐myc vector plasmids or pCMV‐N‐myc‐PIM1. F, indicated the percentage of nuclear/cytoplasmic expression of RUNX3 in E. G, Immunoprecipitation assay showing endogenous cytoplasmic RUNX3 binds with PIM1 in MDA‐MB‐231 and BT‐549 cells. Total cytoplasmic protein was used to detect the direct binding of PIM1 and RUNX3. H, Immunoprecipitation following λ‐protein phosphatase (λ‐PPase) treatment by anti‐PIM1. Note that after λ‐PPase treatment, the PIM1‐RUNX3 complex seemed to disassemble (Lane 4). Data were expressed as means ± SE of three independent experiments. Statistical significance was assessed using unpaired Student's *t* test and one‐way ANOVA (***P < .001; **P < .01; and *P < .05)

Next, we sought to confirm whether PIM1 could regulate RUNX3 subcellular location and whether RUNX3 nuclear dislocation mediates the tumorigenic effects of PIM1. In accordance with previous results, it was shown that RUNX3 dominantly expressed in cytoplasm of TNBC cell line BT‐549 and MDA‐MB‐231, whereas RUNX3 was more localized in the nucleus of MCF‐7 and T47D (Figure S1A‐C). We then sought to interrogate in MCF‐7 and T47D cells whether ectopic PIM1 expression could promote RUNX3 to translocate from nucleus to cytoplasm. The overexpression of PIM1 facilitates ectopic RUNX3‐FLAG nuclear exportation (Figure 5A,B); however, it failed to promote RUNX3(4A)‐FLAG, to translocate from nucleus to cytoplasm (Figure 5C,D). Moreover, PIM1 also failed to facilitate RUNX3(4A) nuclear exportation in MDA‐MB‐231 and BT‐549 cells (Figure 5E,F). Besides, immunoprecipitation confirmed that PIM1 could bind with RUNX3 endogenously in cytoplasm of MDA‐MB‐231 and BT‐549 cells (Figure 5G). Moreover, when cell lysates of MCF‐7 were pre‐treated with protein phosphatase, anti‐PIM1 antibody failed to pull down protein complex containing RUNX3 (Figure 5H), indicating that the phosphorylation status of PIM1 was important for the association between PIM1 and RUNX3. In conclusion, all these results suggested that PIM1 could mediate RUNX3 translocation from nucleus to cytoplasm by binding with and phosphorylating RUNX3.

### 3.5 PIM1 inhibition rescued RUNX3 nuclear expression

**FIGURE 6:**
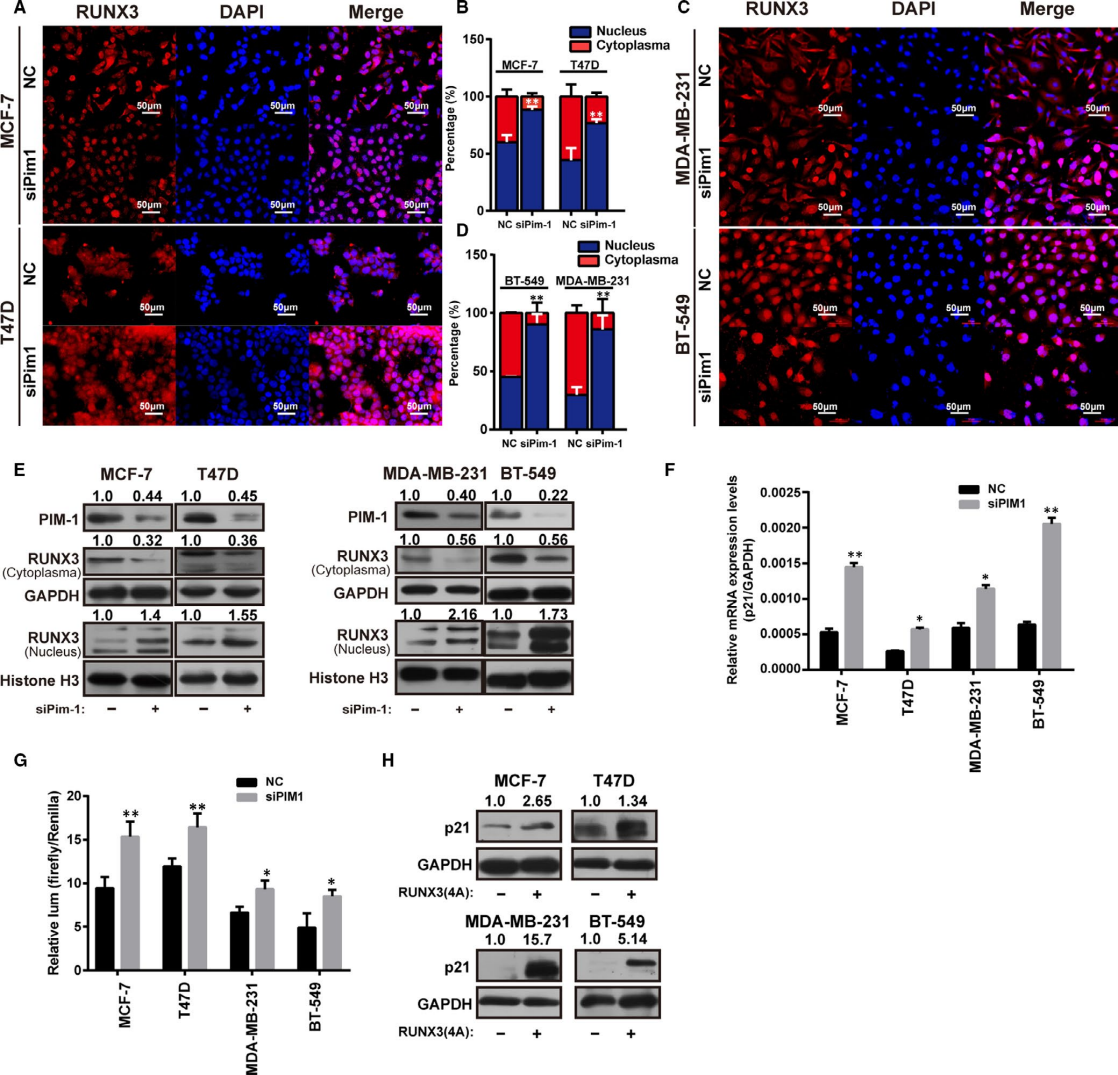
PIM1 knockdown promotes the nuclear relocation of endogenous RUNX3 and its transcriptional activity. A and C, Immunofluorescence images showing the localization of endogenous RUNX3 in MCF‐7, T47D, BT‐549 and MDA‐MB‐231 cells transfected with NC or siPIM1; B, Indicated the percentage of nuclear/cytoplasmic expression of RUNX3 in A, (D) indicated that of RUNX3 in C. E, Western blot analysis showing PIM1 knockdown promoted nuclear expression of endogenous RUNX3 in MCF‐7, T47D, MDA‐MB‐231 and BT‐549 cells. F, Real‐time qPCR analysis showing that PIM1 knockdown promoted the transcription of RUNX3 target gene Cdkn1a (p21) (n = 3). G, Luciferase assay showing the transcriptional activity of Cdkn1a (p21) promoter was improved when knocking down PIM1 (n = 3). H, Western blot analysis showing p21 expression was rescued after overexpressing RUNX(4A). Data were expressed as means ± SE of three independent experiments. Statistical significance was assessed using unpaired Student's *t* test and one‐way ANOVA (***P < .001; **P < .01; and *P < .05)

Then, we sought to interrogate whether PIM1 inhibition could rescue RUNX3 nuclear expression. PIM1 knockdown was shown to promote endogenous RUNX3 to relocate in the nucleus of MCF‐7 and T47D (Figure 6A,B), MDA‐MB‐231 and BT‐549 cells (Figure 6C,D). Moreover, this result was further confirmed by detecting RUNX3 separately in cytoplasmic and nuclear lysates of MCF‐7, T47D, MDA‐MB‐231 and BT‐549 cells transfected with siRNA targeting at PIM1 by Western blot (Figure 6E).

RUNX3 nuclear expression was known to promote RUNX3 target genes transcription. We next sought to examine whether PIM1 knockdown could promote the transcription of RUNX3 target gene Cdkn1a (p21). PIM1 knockdown was shown to promote Cdkn1a (p21) transcription in MCF‐7, T47D, BT‐549 and MDA‐MB‐231 cells (Figure 6F). Moreover, we subcloned Cdkn1a promoter DNA sequence into pGL3‐basic vector, co‐transfected it with siRNA targeting PIM1 or nonsense sequence control (NC) and it was shown that siPIM1 could significantly promote luciferase activity in MCF‐7, T47D, BT‐549 and MDA‐MB‐231 cells, further confirming that PIM1 inhibition could promote RUNX3 target gene transcription (Figure 6G). In line with this, RUNX3(4A) was shown to recapitulate the effects exerted by PIM1 knockdown on Cdkn1a (p21) expression (Figure 6H), which suggested that PIM1 inhibition could promote RUNX3 target genes expression by rescuing RUNX3 nuclear expression.

**FIGURE 7:**
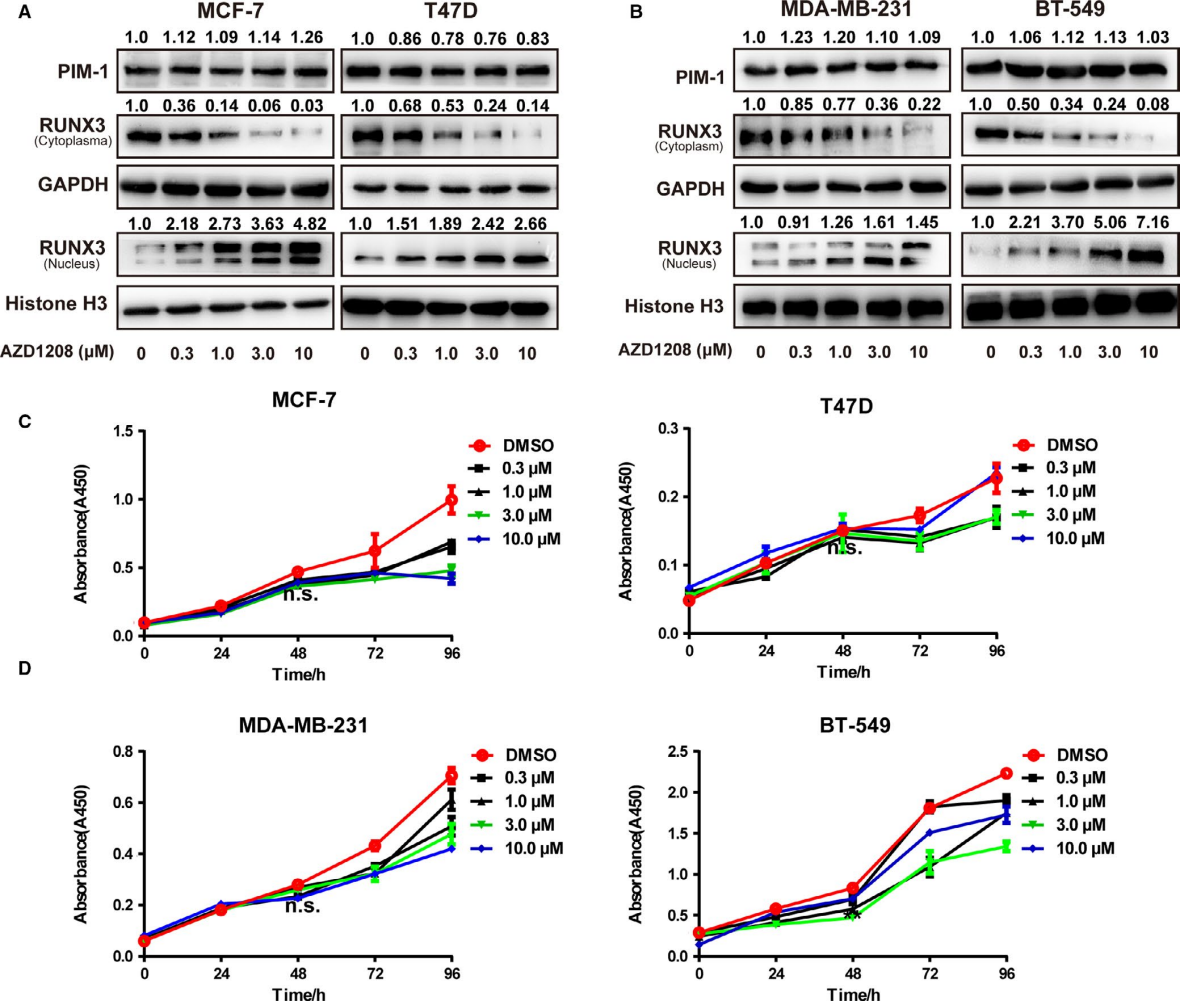
PIM1 inhibitors promoted the nuclear expression of RUNX3. A, Western blot analysis showing AZD1208, the PIM1 kinase inhibitor, reduced the cytoplasmic expression of RUNX3 while promoting its nuclear expression in MCF‐7, T47D (A), MDA‐MB‐231 and BT‐549 (B) cells. (C and D) CCK8 assay showing the effect of AZD1208 treatment on the proliferation rate of MCF‐7, T47D (C) and MDA‐MB‐231, BT‐549 (D) (n = 6). Data were expressed as means ± SE of three independent experiments. Statistical significance was assessed using unpaired Student's *t* test and one‐way ANOVA (***P < .001; **P < .01; and *P < .05)

AZD1208, well‐acknowledged PIM1 inhibitors, was shown to dose dependently promote RUNX3 nuclear expression and inhibit RUNX3 cytoplasmic expression in T47D, MCF‐7, BT‐549 and MDA‐MB‐231 cells (Figure 7A,B), without impairing the cell growth ability within 48 hours (Figure 7C,D). And this further corroborated our hypothesis that inhibiting PIM1 kinase could facilitate RUNX3 nuclear redistribution.

### 3.6 PIM1 facilitates RUNX3 cytoplasmic retention in breast cancer tissues

To validate that PIM1 mediates the cytoplasmic translocation of RUNX3 in breast cancer tissues, we used the aforementioned TMA to conduct an IHC staining of RUNX3 and PIM1. Both whole‐cell or subcellular IHC staining intensity of RUNX3 and PIM1 were scored by experienced pathologists. Total PIM1 was shown to have a marginal‐positive linear correlation with cytoplasmic RUNX3 expression (*r* = .1718, *P* = .012) but a significant negative one with nuclear RUNX3 expression (*r* = −.1817, *P* = .0078). This result strongly suggested that PIM1 could promote the nuclear dislocation of RUNX3 and the positive correlation between PIM1 and cytoplasmic RUNX3 may be ascribed to the translocation effect exerted by PIM1. Moreover, cytoplasmic PIM1 expression had a linear correlation with both cytoplasmic RUNX3 retention (*r* = .2332, *P* = .0006) and nuclear RUNX3 dislocation (*r* = −.2997, *p* < .0001), and we also notice the significantly steeper slope in both circumstances. In our opinions, this implied that the cytoplasmic PIM1 might play a more vital role in the subcellular location of RUNX3.

**FIGURE 8:**
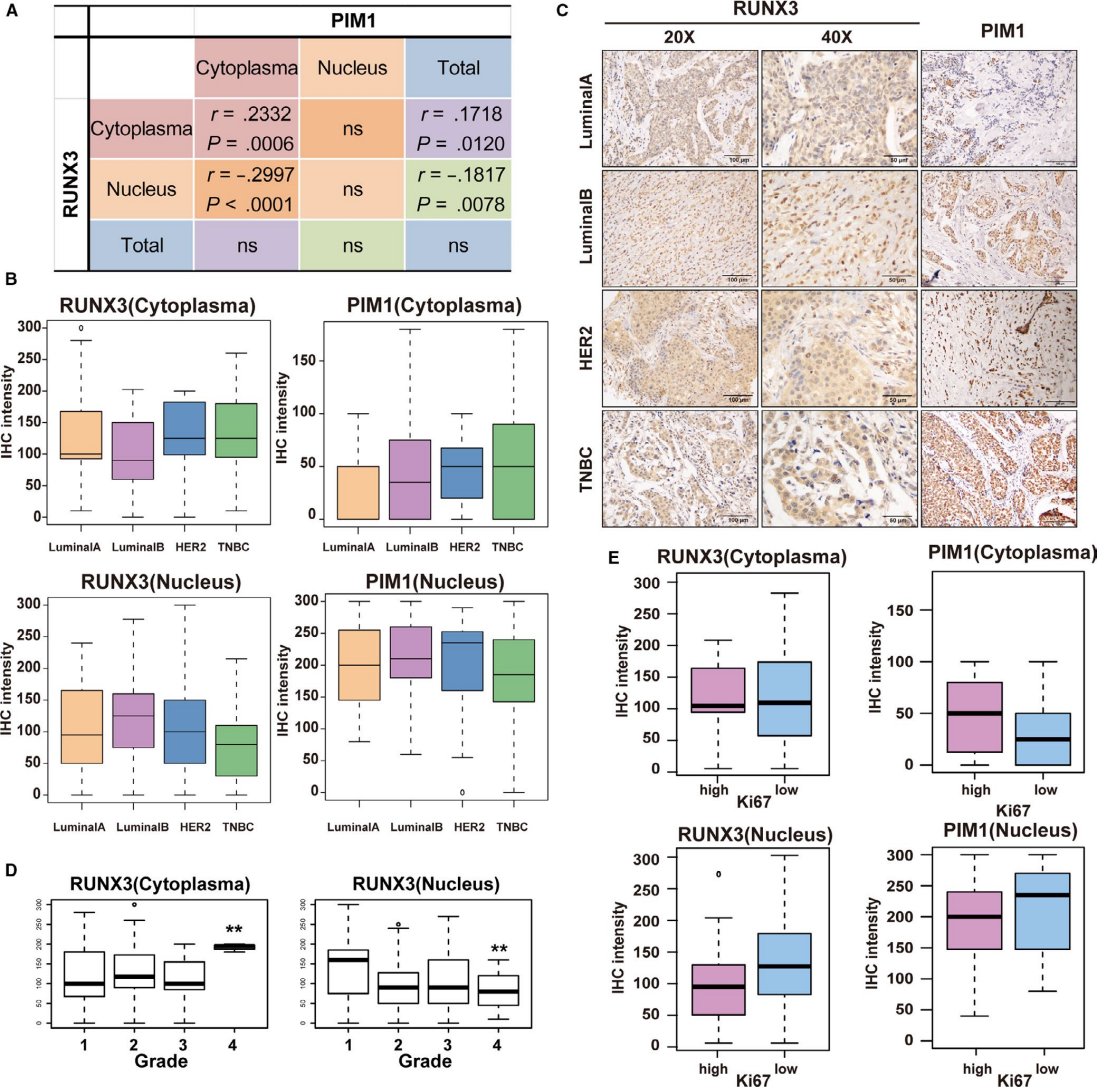
Analysis of the correlation between clinicopathological parameters and PIM1 or RUNX3 in a TMA that consisted of 213 breast cancer cases. A, The table showing the correlation between subcellular or whole‐cell expression of RUNX3 and that of PIM1 by the H‐scores of IHC staining. B, The subcellular RUNX3 IHC intensity in breast cancer tissues with different hormone receptor status. C, Representative images of IHC staining of RUNX3 and PIM1 in breast cancer tissues with different hormone receptor status. D, The cytoplasmic (left) and nuclear (right) RUNX3 staining intensity in breast cancer tissues stratified by histological grades. E, The average subcellular RUNX3 or PIM1 IHC intensity stratified by Ki67 expression. Data were expressed as means ± SE of three independent experiments. Statistical significance was assessed using unpaired Student's *t* test and one‐way ANOVA (***P < .001; **P < .01; and *P < .05)

Next, we sought to further clarify the expression pattern of PIM1 and RUNX3 in breast cancer tissues. It was found that PIM1 co‐localized with RUNX3 in cytoplasm across paraffin tissue section with different hormone receptor status (Figure S3). In accordance with these results, both cytoplasmic RUNX3 expression and PIM1 expression were higher in TNBC cases when compared with that in cases of hormone receptor‐positive types (Figure 8B, upper‐panel); and nuclear RUNX3 and PIM1 expression were lower in TNBC when compared with that in hormone receptor‐positive types (Figure 8B, lower panel). All these results further suggested that PIM1 could facilitate RUNX3 cytoplasmic retention, and this was also confirmed in the representative images of PIM1/RUNX3 IHC staining.

RUNX3 was a well‐acknowledged tumour suppressor as a transcriptional factor. And this was in accordance with our results that cytoplasmic RUNX3 retention and nuclear RUNX3 dislocation was associated with higher histological grade (Figure 8D). Higher nuclear RUNX3 and lower cytoplasmic/higher nuclear PIM1 expression indicated lower Ki67 staining intensity (Figure 8E). All these clinicopathological results suggested that RUNX3 dislocation may mediate the tumorigenic effects of PIM1 in breast cancer.

## 4 DISCUSSION

Pim oncogenes are overexpressed in a wide range of tumours from a haematological or epithelial origin.^4^ The mechanisms by which PIM1 exerted its oncogenic activity were reviewed by predecessors and are boiled down to three major aspects: through the regulation of MYC; through the regulation of cap‐dependent protein translation; and through the regulation of cell cycle progression. Here, in our work, we extended our knowledge of PIM1 as an oncogene, by revealing the possible role of PIM1 in regulating the BrCSC‐like traits of breast cancer cells. To be specific, we confirmed that PIM1 could phosphorylate RUNX3 to promote its translocation from nucleus to cytoplasm; PIM1 inhibition, either by knocking down PIM1 or kinase inhibitor, could attenuate the BrCSC‐like traits of breast cancer cells; RUNX3 were confirmed to mediate the anti‐BrCSC effects of PIM1 inhibition. In this way, we also broaden our knowledge of RUNX3 as a tumour suppressor, and why its inadequate expression in nuclei contributes to tumour progression and relapse.

RUNX3 was reported to be phosphorylated by a spectrum of other oncogenic kinases, like Pak1, Pin and Srk. And in triple‐negative breast cancer, those pro‐tumour kinases are pervasively overactivated to maintain the uncontrolled growth of tumour cells. This may well explain the observed nuclear dislocation of RUNX3 in TNBC human tissues and cell lines as well, where RUNX3 might act as a sensor of the intricate pro‐growth kinases signaling.^18^, ^19^, ^20^ As TNBC is generally deemed to be more invasive, recalcitrant to chemotherapeutic agents and more likely to relapse, RUNX3 and its upstream kinases PIM1 promise to be a druggable target for eradicating TNBC.

A TMA consisting of 213 breast cancer patients was included in our research. As to our knowledge, this is the first comprehensive research of PIM1 expression across breast cancer tissues with different hormone receptors status. In line with published work, high PIM expression was shown to be connected with RUNX3 nuclear dislocation and cytoplasmic retention.^23^, ^28^ Interestingly, PIM1 cytoplasmic expression seemed to correlate more closely with RUNX3 subcellular location when compared with nuclear or whole‐cell PIM1 expression. TNBC tissue sections claimed more intensified expression of cytoplasmic PIM1 and RUNX3 expression, highlighting the oncogenic role of cytoplasmic PIM/RUNX3. Also, it remains an open question whether cytoplasmic RUNX3 holds some oncogenic effects, since to date no publication refers to the possible pro‐tumour role of cytoplasmic RUNX3, independent of the loss of its tumour‐suppressor effects due to nuclear dislocation. And this may well explain the two‐edge role of RUNX3 in different tumours, because it seems like that RUNX3 functions as an oncogenic factors in some rare cases.^29^, ^30^, ^31^, ^32^ RUNX3 cytoplasmic retention was already reported by other group to be associated with poor prognosis in colorectal cancer.^33^ Here, in this work, our group also reported this phenomenon that cytoplasmic RUNX3 retention indicated poor prognosis in breast cancer, further highlighting the possible oncogenic role of cytoplasmic RUNX3. It also reminded us that the comprehensive character RUNX3 played in tumour biology may due to the cellular context of both extrinsic and intrinsic kinases profile, and PIM1 might be one of the most important regulators of RUNX3 subcellular location. However, how to interrogate the cytoplasmic RUNX3 performance alone needs more sophisticatedly designed experiments. Besides, PIM1 was shown by our results to co‐localize with and directly bind with RUNX3 in cytoplasm of breast cancer cells. However, Ionov Y *et al* reported that PIM1 protein kinase is nuclear in Burkitt's lymphoma and nuclear localization is necessary for its biological effects.^34^ It seems that whether subcellular localization of PIM1 makes a difference still remains controversial. This prompted us to further investigate whether cytoplasmic exerts oncogenic effects by detaining PIM1 or the other way round.

RUNX3 was referred by other groups to mediate the epithelial‐mesenchymal transition induced by TGF‐β and play a part in the stem cell–like traits induced by aberrantly activated Wnt signalling in gastric cancer^35^ and to reduce cancer stem cells in hepatocellular carcinoma by suppressing Jagged1‐Notch signalling.^36^ However, the role of RUNX3 in breast cancer stem cell was barely mentioned. We confirmed in our work that RUNX3 could attenuate the stem cell–like traits in breast cancer cells and mediate the antitumour effects of PIM1 inhibition in this way. We also firstly reported the “pro‐stemness” effects of PIM1 in breast cancer cells. In consideration of the fact that stem–like traits were intricately correlated with tumour metastasis, relapse and resistance to chemical agents,^37^, ^38^ we suggested that PIM1 could promote tumour metastasis by facilitating RUNX3 nuclear dislocation. More work needed to be conducted to interrogate whether PIM1 inhibition could suppress tumour metastasis in breast cancer. Targeting cancer stem cells (CSCs) population was for a long time being a research hot spot.^37^ However, due to the ever‐changing tumour micro‐environment (TME) and intrinsic tumour biology, CSC population within tumour bulk is hardly druggable. To find out the “driver gene” in CSC population is important for developing new breast cancer treatment strategies. And in this paper, we revealed PIM1 to be promising for targeting at to wipe out CSC population specifically due to its regulation on RUNX3 cytoplasmic translocation, and inhibition of PIM1 could attenuate stem cell–like traits of breast cancer cells.

In conclusion, our work demonstrated that PIM1 could site‐specifically phosphorylate RUNX3 to promote its translocation from nucleus to cytoplasm by protein‐protein interaction. And we also revealed PIM1 inhibition as a new strategy to target at cancer stem cells population and RUNX3 was essential to the anti‐BrCSC effect of PIM1 inhibition.

## CONFLICT OF INTEREST

The authors declared that they have no conflicts of interest to this work.

## AUTHOR CONTRIBUTION

CX and HL designed and conducted most of the experiments. QY, YL and CC conducted the TMA construction and the IHC experiments. CX, CC, QL and YL conducted the plasmid constructions. CX, HL and DM analysed the data and wrote the manuscript.

## ETHICAL APPROVAL

The study was approved by the Ethics Committee of the First Affiliated Hospital of Xuzhou Medical University; informed consent was waived for this study.

## Supporting information

Figure S1

Figure S2

Figure S3

## Data Availability

The data that support the findings of this study are available from the corresponding author upon reasonable request.
