# Supplementary figures and images for: Inhibition of PIM1 attenuates the stem cell–like traits of breast cancer cells by promoting RUNX3 nuclear retention
Source: J Cell Mol Med. 2020 Apr 19;24(11):6308–23. doi: 10.1111/jcmm.15272 (PMC7294145; doi:10.1111/jcmm.15272)

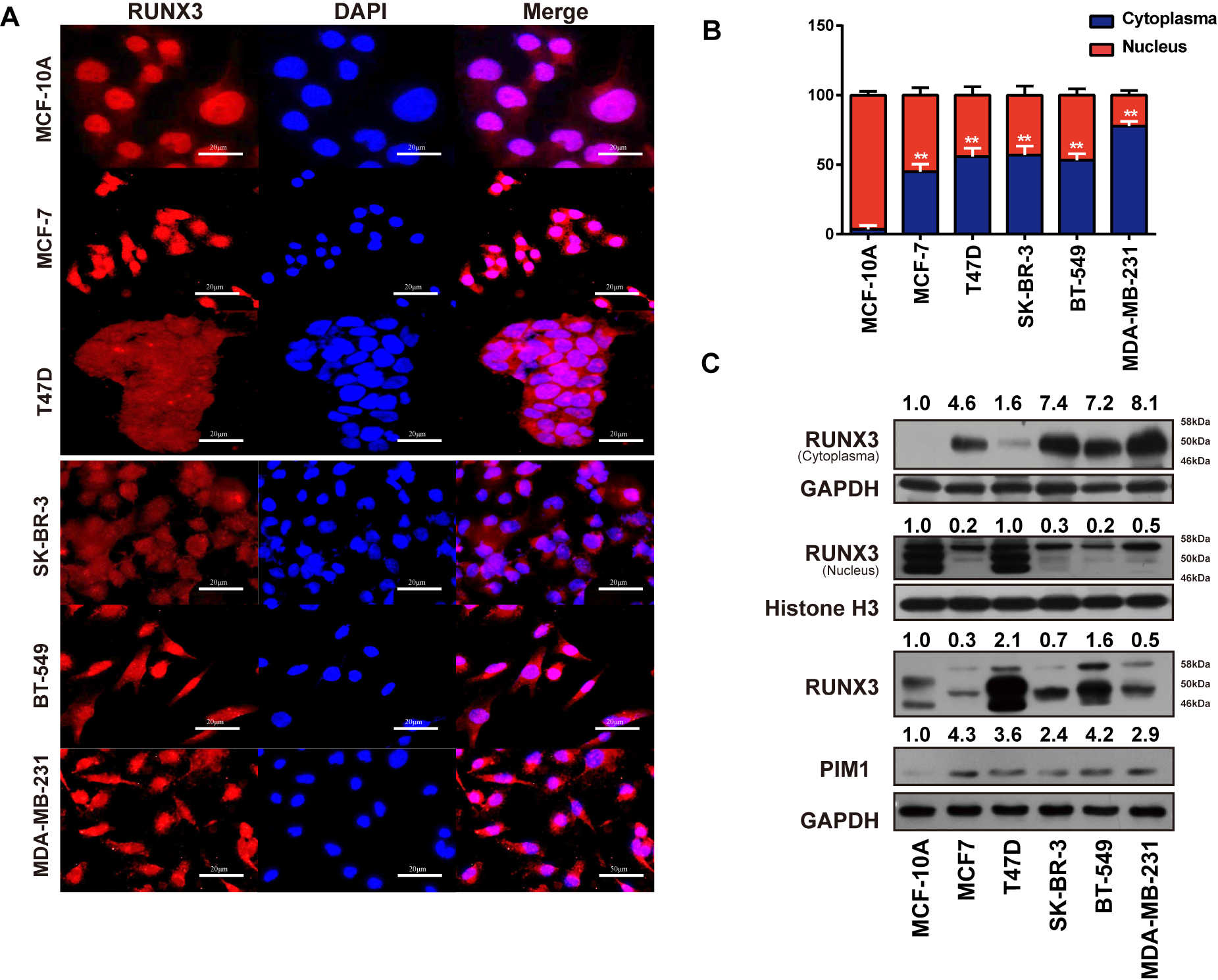

Supplement: Supplementary file 1 — Figure S1 [file JCMM-24-6308-s001.tif]

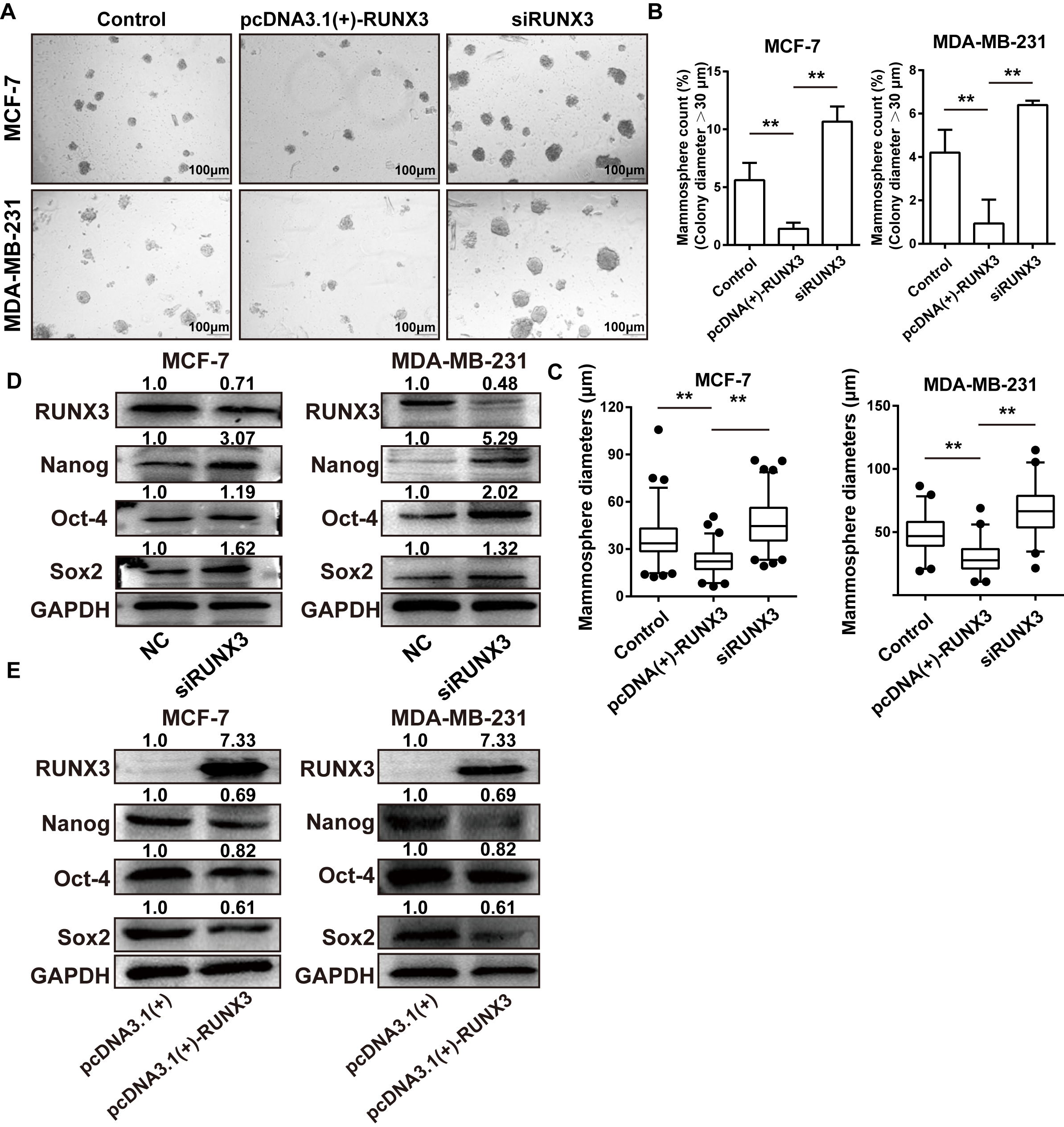

Supplement: Supplementary file 2 — Figure S2 [file JCMM-24-6308-s002.tif]

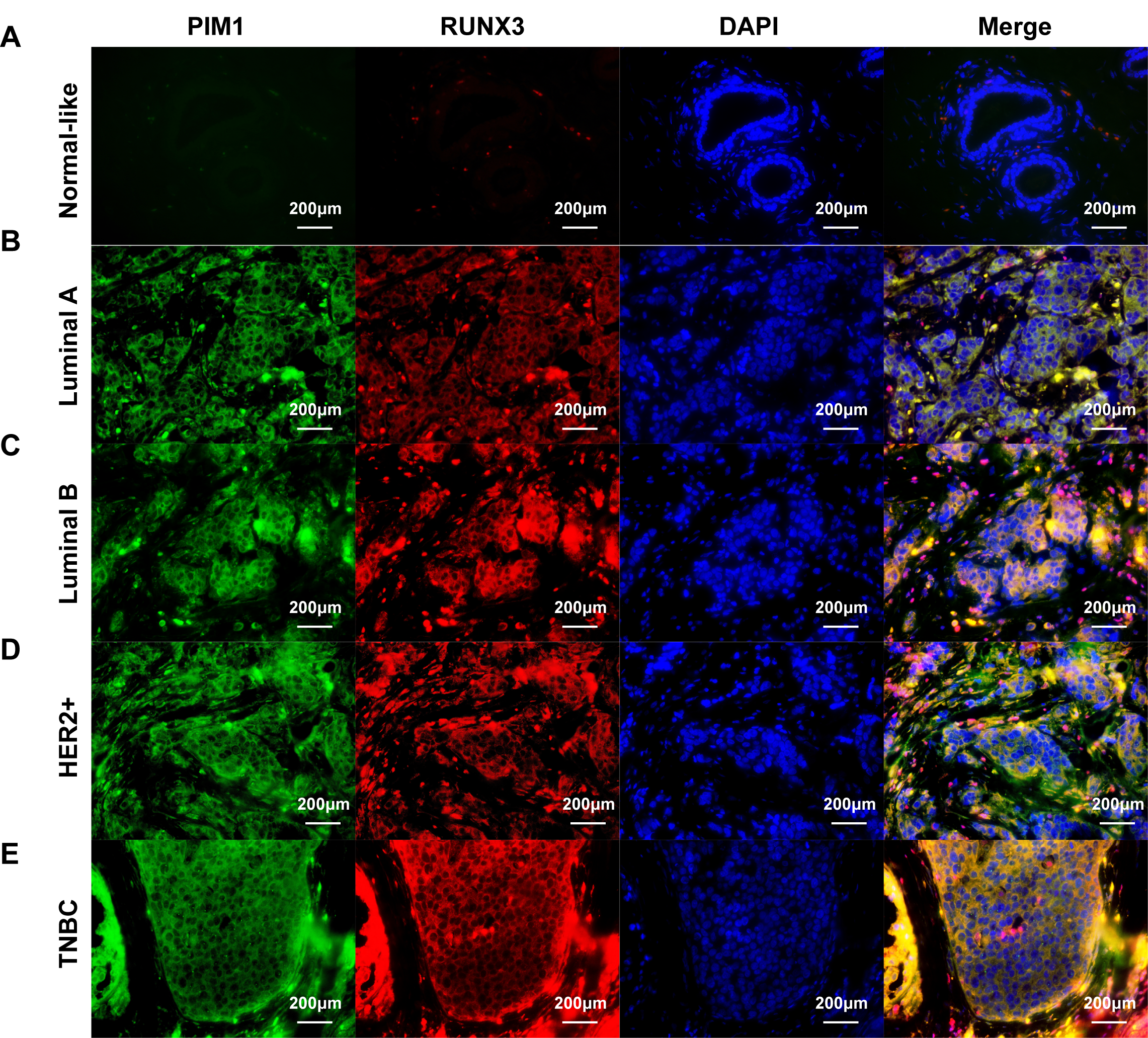

Supplement: Supplementary file 3 — Figure S3 [file JCMM-24-6308-s003.tif]
